# Interleukin-6 Induces Myogenic Differentiation via JAK2-STAT3 Signaling in Mouse C2C12 Myoblast Cell Line and Primary Human Myoblasts

**DOI:** 10.3390/ijms20215273

**Published:** 2019-10-24

**Authors:** Paul J. Steyn, Kevin Dzobo, Robert I. Smith, Kathryn H. Myburgh

**Affiliations:** 1 Department of Physiological Sciences and Biochemistry, Stellenbosch University, Matieland 7602, Stellenbosch, South Africa; paul.steyn@uct.ac.za (P.J.S.); kdzobosnr@yahoo.com (K.D.); robs@smmafrica.com (R.I.S.); 2 International Centre for Genetic Engineering and Biotechnology (ICGEB), Cape Town Component and Institute of Infectious Disease and Molecular Medicine, Wernher and Beit Building (South), University of Cape Town Medical Campus, Anzio Road, Observatory 7925, Cape Town, South Africa

**Keywords:** muscle, myogenesis, satellite cells, myoblasts, IL-6, interleukins, JAK-STAT signaling, differentiation, proliferation, regeneration

## Abstract

Postnatal muscle growth and exercise- or injury-induced regeneration are facilitated by myoblasts. Myoblasts respond to a variety of proteins such as cytokines that activate various signaling cascades. Cytokines belonging to the interleukin 6 superfamily (IL-6) influence myoblasts’ proliferation but their effect on differentiation is still being researched. The Janus kinase (JAK)-signal transducer and activator of transcription (STAT) pathway is one of the key signaling pathways identified to be activated by IL-6. The aim of this study was to investigate myoblast fate as well as activation of JAK-STAT pathway at different physiologically relevant IL-6 concentrations (10 pg/mL; 100 pg/mL; 10 ng/mL) in the C2C12 mouse myoblast cell line and primary human myoblasts, isolated from eight young healthy male volunteers. Myoblasts’ cell cycle progression, proliferation and differentiation in vitro were assessed. Low IL-6 concentrations facilitated cell cycle transition from the quiescence/Gap1 (G0/G1) to the synthesis (S-) phases. Low and medium IL-6 concentrations decreased the expression of myoblast determination protein 1 (MyoD) and myogenin and increased proliferating cell nuclear antigen (PCNA) expression. In contrast, high IL-6 concentration shifted a larger proportion of cells to the pro-differentiation G0/G1 phase of the cell cycle, substantiated by significant increases of both MyoD and myogenin expression and decreased PCNA expression. Low IL-6 concentration was responsible for prolonged JAK1 activation and increased suppressor of cytokine signaling 1 (SOCS1) protein expression. JAK-STAT inhibition abrogated IL-6-mediated C2C12 cell proliferation. In contrast, high IL-6 initially increased JAK1 activation but resulted in prolonged JAK2 activation and elevated SOCS3 protein expression. High IL-6 concentration decreased interleukin-6 receptor (IL-6R) expression 24 h after treatment whilst low IL-6 concentration increased IL-6 receptor (IL-6R) expression at the same time point. In conclusion, this study demonstrated that IL-6 has concentration- and time-dependent effects on both C2C12 mouse myoblasts and primary human myoblasts. Low IL-6 concentration induces proliferation whilst high IL-6 concentration induces differentiation. These effects are mediated by specific components of the JAK/STAT/SOCS pathway.

## 1. Introduction

Muscle satellite cells are adult progenitor cells formed during vertebrate embryonic development [1,2,3]. Adult satellite cells are quiescent cells found between the plasma membrane of myofibers and the basal lamina and express several marker proteins such as paired box protein 7 (Pax7), cluster of differentiation 34 (CD34), desmin and M-cadherin [4,5,6,7]. Satellite cells are associated with muscle growth after child-birth [8,9,10]. The loss of muscle fibers, due to old age and diseases, results in blunted functional capacity as well as morbidity [11,12]. Cachexia, an extreme form of wasting of the body, is a symptom of chronic illness and causes severe muscle loss [13]. Exercise and injury activate Pax7^+^ muscle satellite cells [14,15,16]. The myogenic regulatory factors (MRFs) expressed by satellite cells are required for muscle regeneration and include myoblast determination protein 1 (MyoD) [4,5,6,7]. Pax7 maintains satellite cell self-renewal [14,16,17,18]. MyoD expressing satellite cells exit the cell cycle and differentiate to form multinucleated myocytes in order to repair damaged muscles [19,20,21]. Several lines of evidence show that myoblasts contribute significantly to muscle regeneration in vivo [22,23,24,25,26,27,28].

Several myogenic cell lines derived from rodents’ muscle satellite cells have been utilized in studying muscle progenitor cell proliferation and differentiation [29]. These cell lines include C2C12 and L6, with C2C12 providing an excellent model to study myoblasts’ proliferation and differentiation [30,31,32,33,34]. The regulation of myogenesis is controlled by transcription factors. Early responders specific to muscle are classified as MRFs and include Myf5, MyoD, MRF4 and myogenin [35,36,37]. Dimerization of MRFs with gene *E2A* products results in the activation of many myogenesis-specific genes [38,39,40,41,42]. Expression of myogenin is a signal that myoblasts have committed to differentiation as the process can no longer be reversed [43,44]. Another family of transcription factors is the myocyte enhancer-binding factor 2 (MEF2) that also contributes to maturation of differentiating myoblasts. 

The mechanism(s) involved in the regulation of satellite proliferation and differentiation have received a lot of attention although some aspects are still unclear [14,15,45]. Several growth factors and cytokines, including leukemia inhibitory factor (LIF), transforming growth factor (TGF-β) and hepatocyte growth factor (HGF), have been implicated as key in these processes [46,47,48,49,50,51]. Research into the precise roles that different concentrations of these satellite cell regulators play in these processes is still an active area of research. Previously considered an inflammatory cytokine, interleukin-6 (IL-6) is now known to be both a cytokine produced by a variety of cell types and a myokine produced by muscle cells [49,52,53,54]. Several lines of evidence from in vivo experiments have delineated the importance of IL-6 and the activation of downstream signaling. IL-6 knockout results in reduced hypertrophic response in rodents [48,55], suggesting a role in differentiation. However, these studies also reported that one of the consequences of IL-6 knockout was reduced satellite cell proliferation as a result of loss of signal transducer and activator of transcription (STAT3) signaling compared to controls [48,55]. Molecular details of the binding of IL-6 to its receptor and the resultant STAT3 phosphorylation via the Janus kinase (JAK)-2 have been described before [56]. Trenerry and colleagues showed that STAT3 is activated in human skeletal muscle after bouts of exercise in healthy young volunteers [57]. The activation of STAT proteins through the binding of ligands such as IL-6 is a transient process with the effect seen within minutes to several hours [58]. IL-6 levels and satellite cell numbers increased significantly in human muscle biopsies after an acute bout of muscle damaging physical exercise, suggesting that IL-6 was involved in satellite cells’ proliferation [52,59]. Detailed in vitro experiments have revealed that phosphorylated STAT3 translocates to the nucleus where it promotes transcription of several genes [58,60]. IL-6 and phosphorylated STAT3-induced genes have been linked with cell cycle regulation and proliferation [48]. A feedback loop exists whereby STAT3 regulates its upstream partners such as IL-6, suppressor of cytokine signaling 3 (SOCS3) and interleukin-6 receptor [61,62]. In addition, STAT3 has been found to display context-dependent influences on several processes including proliferation and differentiation [63,64].

The mechanism by which IL-6 influences the STAT3 signaling cascade is by binding to the transmembrane gp130 receptor and the subsequent activation of JAK-STAT pathway [65,66]. Previous studies of IL-6 signaling showed that both the classic and trans-signaling mechanisms are in operation [67,68,69]. The IL-6 receptor can exist in membrane-bound or soluble forms and both forms bind to IL-6 with the same affinity. When IL-6 binds to the soluble IL-6 receptor (IL-6R) the process is referred to as trans-signaling [70,71]. The soluble IL-6R is found in body fluids [69,72]. The IL-6-IL-6R complex formed also binds to the gp130 homodimer, in a way amplifying the activities and effect of IL-6, by inducing signaling using two mechanisms [69,72,73]. The JAK-STAT cascade consists of JAK1, JAK2, JAK3 and Tyk2 whilst there are seven STATs, STAT1-4, STAT5a, STAT5b and STAT6 [66,74]. The JAK-STAT cascade has been identified as important for proliferation in many cell types including skeletal muscle in vitro [45,75] and in vivo [52,59]. Several studies have shown the involvement of JAK-STAT cascade in myoblast proliferation in rat muscles and in in vitro cultured primary myoblasts [49,76,77]. Whilst several studies were done on rodents, differences between rodents and humans exists [78,79,80]. For in vivo experiments, there is little control of concentrations of cytokines. Therefore, very little evidence is available on how the JAK-STAT pathway responds to different concentrations of IL-6 under various conditions. Several studies including those by Kurosaka and Machida have used IL-6 concentrations within the range used in this study [59,72,81,82]. Whilst the phosphatidylinositol-3 kinase/protein kinase B (PI3K-Akt) and the p38 mitogen-activated kinase (p38 MAPK) are known to positively regulate myogenic differentiation, the role of JAK-STAT cascade in IL-6-mediated myoblast differentiation has not been investigated.

Therefore, the aim of this study was to evaluate the effect of physiologically relevant IL-6 concentrations on myoblasts’ proliferation, differentiation and the activation of the JAK-STAT pathway. Proliferating myoblasts, either C2C12 or of primary human origin, were treated with different IL-6 concentrations, and cell behavior at different time points was investigated. Based on our data, the main findings were that low IL-6 concentration induced proliferation of myoblasts as shown by increased proliferation markers such as proliferating cell nuclear antigen (PCNA)through JAK1 activation and feedback through SOCS1 expression. High IL-6 induced differentiation of myoblasts as shown by increased myogenin expression through JAK2 activation and increased SOCS3 expression. Increases in IL-6R at low IL-6 concentration were observed, possibly serving as a compensatory mechanism to increase binding of IL-6 to its receptor to increase signaling. At high IL-6, the decrease in IL-6R observed may serve a homeostatic role to reduce IL-6 signaling. Overall, these data imply that the concentration of IL-6 plays a major role in determining whether activated satellite cells respond to IL-6 by proliferating more or differentiating. Furthermore, a feedback loop exists whereby negative regulators such as SOCS1 and SOCS3 can abrogate the effect of IL-6-mediated increase in JAK-STAT signaling.

## 2. Results

### 2.1. Effect of IL-6 on C2C12 Cell Cycling is Mediated via the JAK-STAT Signaling Cascade

**Figure 1:**
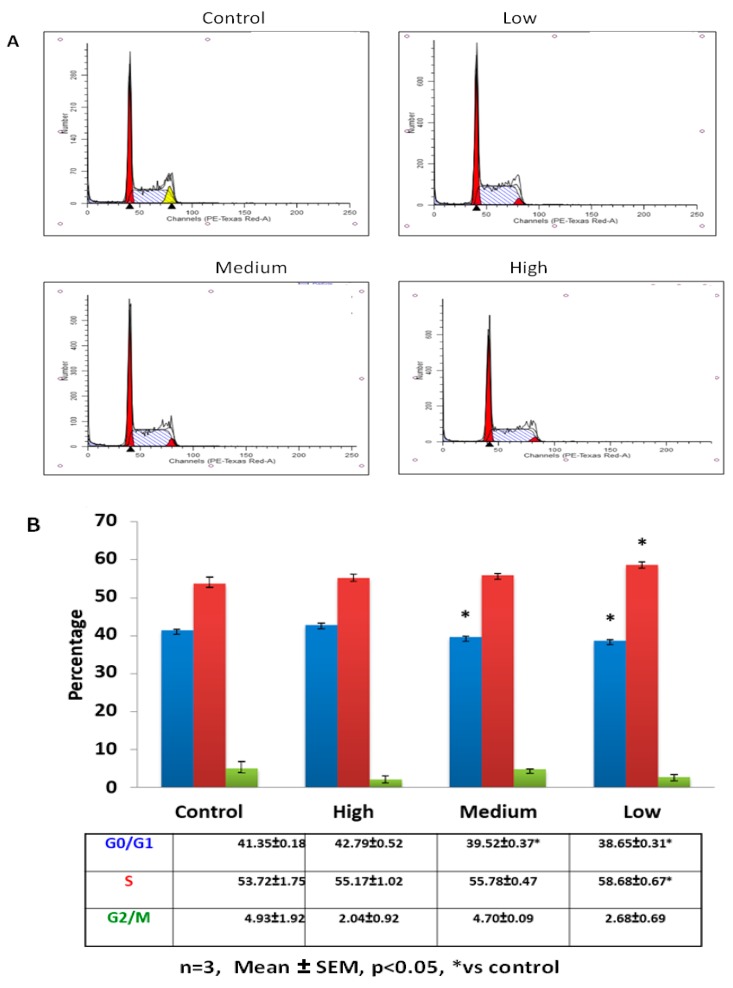
Effect of low, medium and high IL-6 concentrations on C2C12 cell cycle progression at 24 h. (**A**) Flow cytometric analysis of IL-6-treated cells to determine the effect of IL-6 concentration on C2C12 cell cycling was done after 24 h incubation (**B**) C2C12 cells (%) in each cell cycle stage after treatment of cells with low, medium and high IL-concentrations for 24 h. The experiment was repeated three times. Results are presented as mean ± standard error of the mean (S.E.M). Statistical analysis: Analysis of variance (ANOVA) and Bonferroni post hoc test. * *p* < 0.05; relative to control.

**Figure 2:**
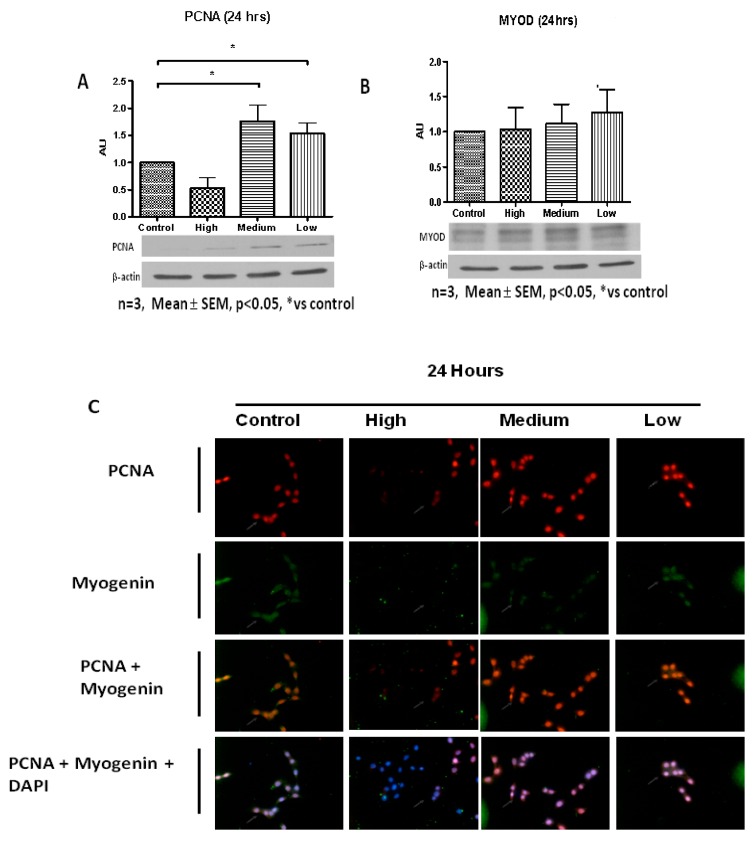
Effect of low, medium and high IL-6 concentrations on PCNA, MyoD and myogenin protein levels in C2C12 cells. (**A**) PCNA protein levels in C2C12 cells treated with low, medium and high IL-6 concentrations for 24 h. (**B**) MyoD protein levels in C2C12 cells treated with low, medium and high IL-6 concentrations for 24 h. (**C**) Immunofluorescence staining for PCNA and myogenin in C2C12 cells treated with low, medium and high IL6 concentrations for 24 h. The experiment was repeated three times. Immunofluorescence images were taken at 100× magnification. Results are presented as mean ± standard error of the mean (S.E.M). Statistical analysis: ANOVA and Bonferroni post hoc test. * *p* < 0.05.

The putative role of IL-6 on C2C12 immortalized muscle cell cycling was investigated using flow cytometry. Control and IL-6-treated cells exhibited significant differences in cell cycle profiles 24 h after treatment (Figure 1A,B). C2C12 cells treated with low (10 pg/mL) and medium (100 pg/mL) IL-6 concentrations displayed similar cell cycle profiles, albeit some small but not significant variations. Both low and medium IL-6 concentrations resulted in decreased number of cells in quiescence/Gap1 (G0/G1) phase compared to control, with the low IL-6-treated cells increasing the proportion in synthesis (S)-phase compared to controls (Figure 1A,B). In contrast, cells treated with high IL-6 concentration displayed similar cell cycle profiles to controls (Figure 1A,B). Furthermore, low and medium IL-6 concentrations significantly induced PCNA protein levels compared to controls (Figure 2A, for level of significance see Figure legends). High IL-6-treated cells had similar PCNA protein levels compared to control cells (Figure 2A). In addition, MyoD protein levels were similar to controls for all IL-6 concentrations (Figure 2B). Immunofluorescence staining substantiated the protein results, displaying increased PCNA levels when C2C12 cells were treated with low and medium IL-6 concentrations for 24 h (Figure 2C). Similar to MyoD, myogenin protein was not altered (Figure 2C).

**Figure 3:**
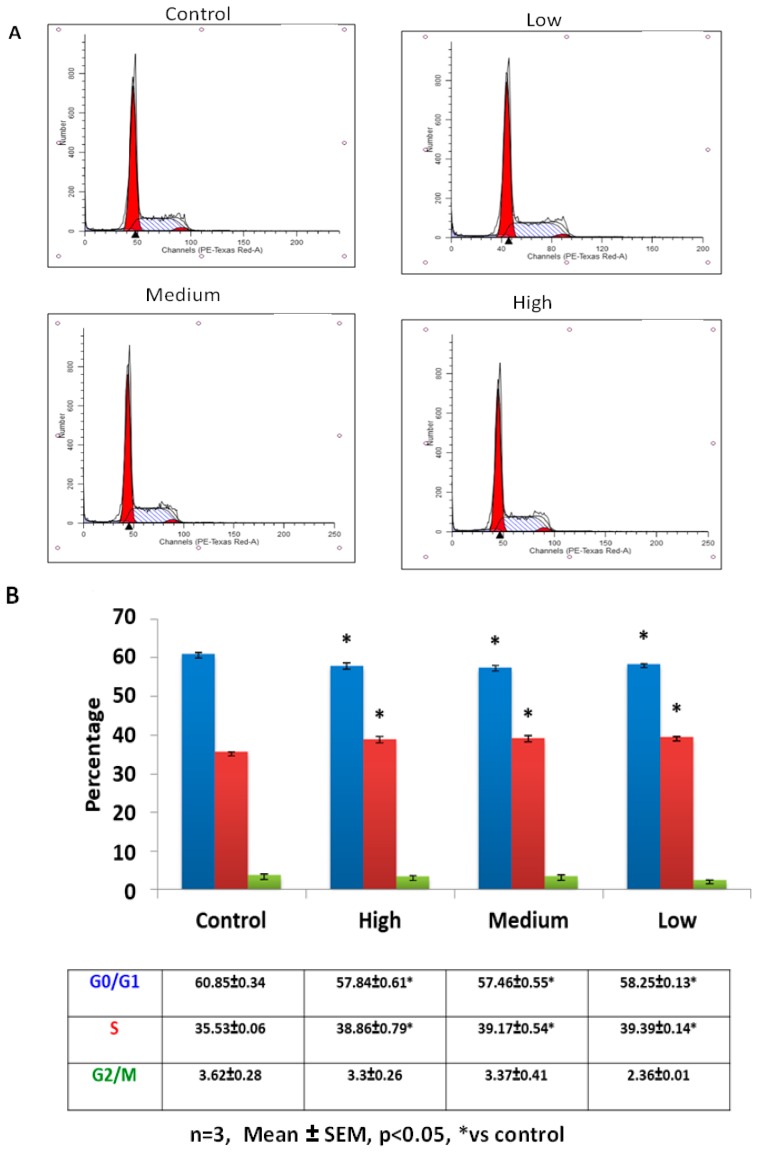
Effect of low, medium and high IL-6 concentration on C2C12 cell cycling after 48 h of treatment. (**A**) Flow cytometric analysis of IL-6-treated cells to determine the effect of low, medium and high IL-6 concentrations on C2C12 cell cycling was done after 48 h incubation (**B**) C2C12 cells (%) in each cell cycle stage after treatment of cells with low, medium and high IL-6 concentrations for 48 h. The experiment was repeated three times. Results are presented as mean ± standard error of the mean (S.E.M). Statistical analysis: ANOVA and Bonferroni post hoc test. * *p* < 0.05; relative to control.

**Figure 4:**
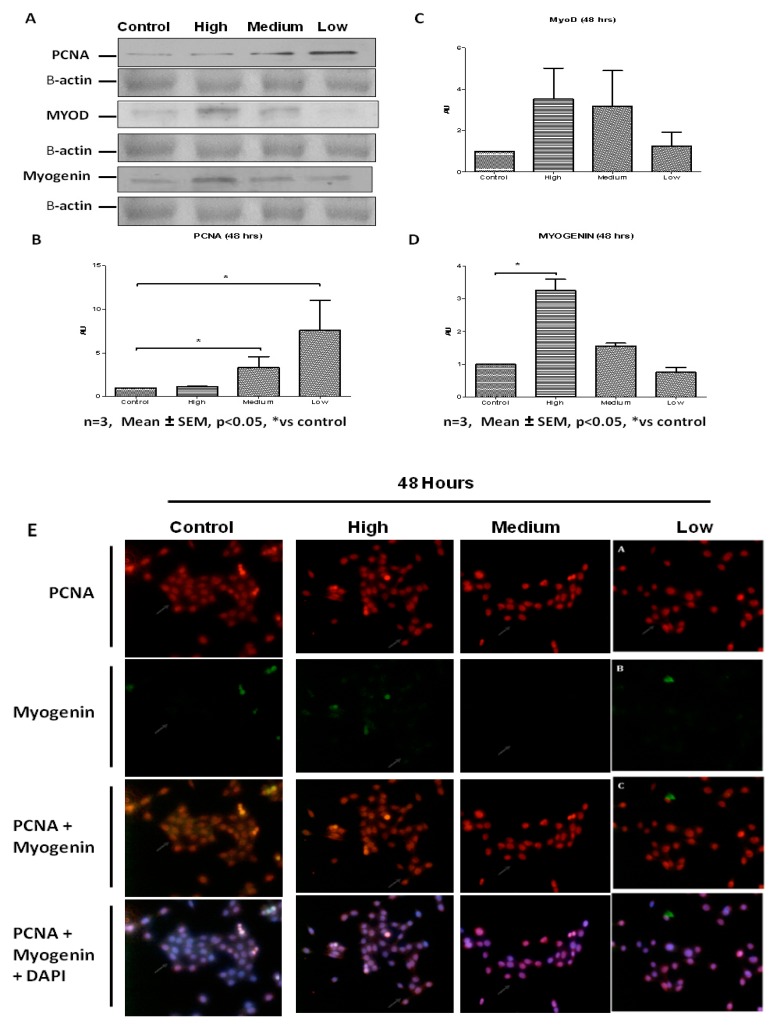
Concentration-dependent effect of IL-6 on C2C12 myogenic regulatory factors’ protein levels. (**A**) Evaluation of PCNA, MyoD and myogenin protein levels in C2C12 cells treated with low, medium and high IL-6 concentrations for 48 h. (**B**) Quantification of PCNA protein levels as shown in (**A**). (**C**) Quantification of MyoD protein levels as shown in (**A**). (**D**) Quantification of myogenin protein levels as shown in (**A**). (**E**) Immunofluorescence staining for PCNA and myogenin in C2C12 cells after treatment with low, medium and high IL-6 concentrations for 48 h. The experiment was repeated three times. Immunofluorescence images were taken at 100× magnification. Results are presented as mean ± standard error of the mean (S.E.M). Statistical analysis: ANOVA and Bonferroni post hoc test. * *p* < 0.05.

Addition of IL-6 for 48 h resulted in C2C12 cells responding in similar fashion to both low and medium IL-6 concentrations (Figure 3A,B). Low and medium IL-6 concentrations decreased the number of cells in the G0/G1 phases whilst increasing those in the S-phase (Figure 3A,B). Western blot analysis showed PCNA levels were increased when C2C12 cells were treated with low and medium IL-6 concentrations (Figure 4A,B). Immunofluorescence analysis substantiated these findings, with C2C12 cells showing increased expression of PCNA when cells were treated with low and medium IL-6 concentrations (Figure 4E). Whilst changes in MyoD were not statistically significant, treatment of cells with high IL-6 concentration resulted in increased myogenin levels (Figure 4A,C,D). This response to high IL6 concentration was accompanied by no effect on PCNA levels, which remained similar to controls (Figure 4A,B). The immunofluorescence data reinforced these findings, clearly demonstrating that high IL-6 concentrations favor myogenic differentiation of C2C12 cells whilst low and medium IL-6 concentrations induce C2C12 proliferation (Figure 4A,E).

**Figure 5:**
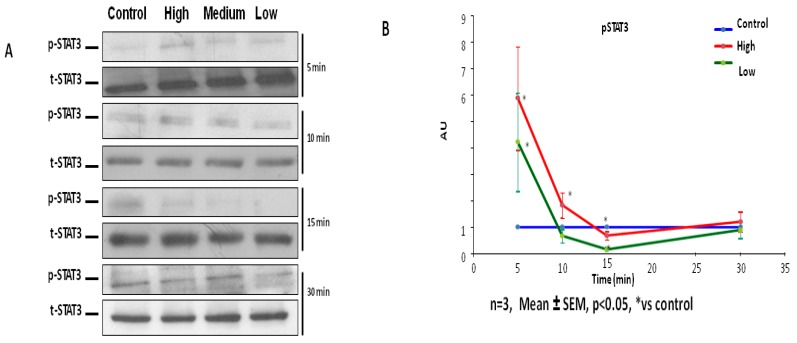

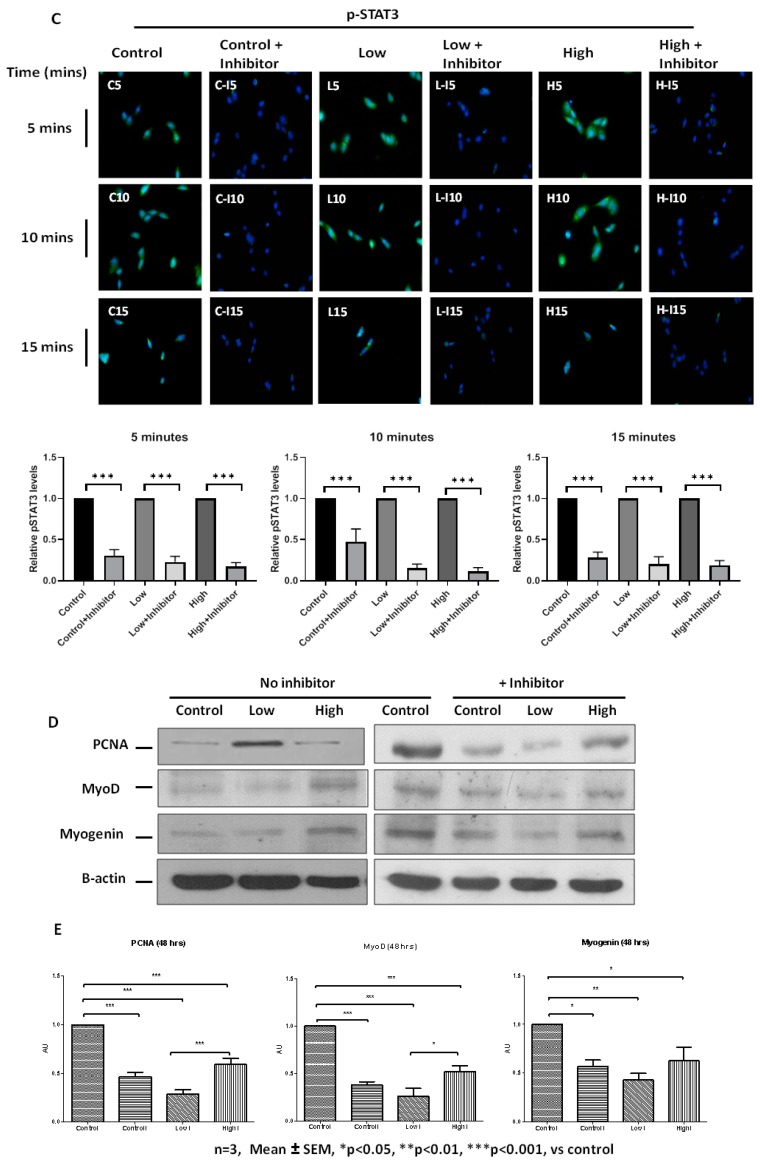
IL-6 activates the STAT3 signaling pathway in C2C12 cells. (**A**) Evaluation of p-STAT3 protein levels in C2C12 cells at 5 min up to 30 min after treatment of cells with low, medium and high IL-6 concentrations. (**B**) Quantification of western blot images shown in (**A**)**.** (**C**) Effect of JAK-STAT inhibitor, AG490, on p-STAT3 expression in C2C12 cells over 15 min after treatment with low and high IL-6 concentration. (**D**) Effect of JAK-STAT inhibitor, AG490, on PCNA, MyoD and myogenin protein levels in C2C12 cells after treatment with low and high IL-6 concentrations (top panel) and densitometric quantification of images (lower panel) (**E**) Quantification of PCNA, MyoD and myogenin protein levels in C2C12 cells after treatment with low and high IL-6 concentrations plus JAK-STAT inhibitor, AG490. The experiment was repeated three times. Immunofluorescence images were taken at 100× magnification. Results are presented as mean ± standard error of the mean (S.E.M). Statistical analysis: ANOVA and Bonferroni post hoc test. * *p* < 0.05; ** *p* < 0.01; *** *p* < 0.001.

**Figure 6:**
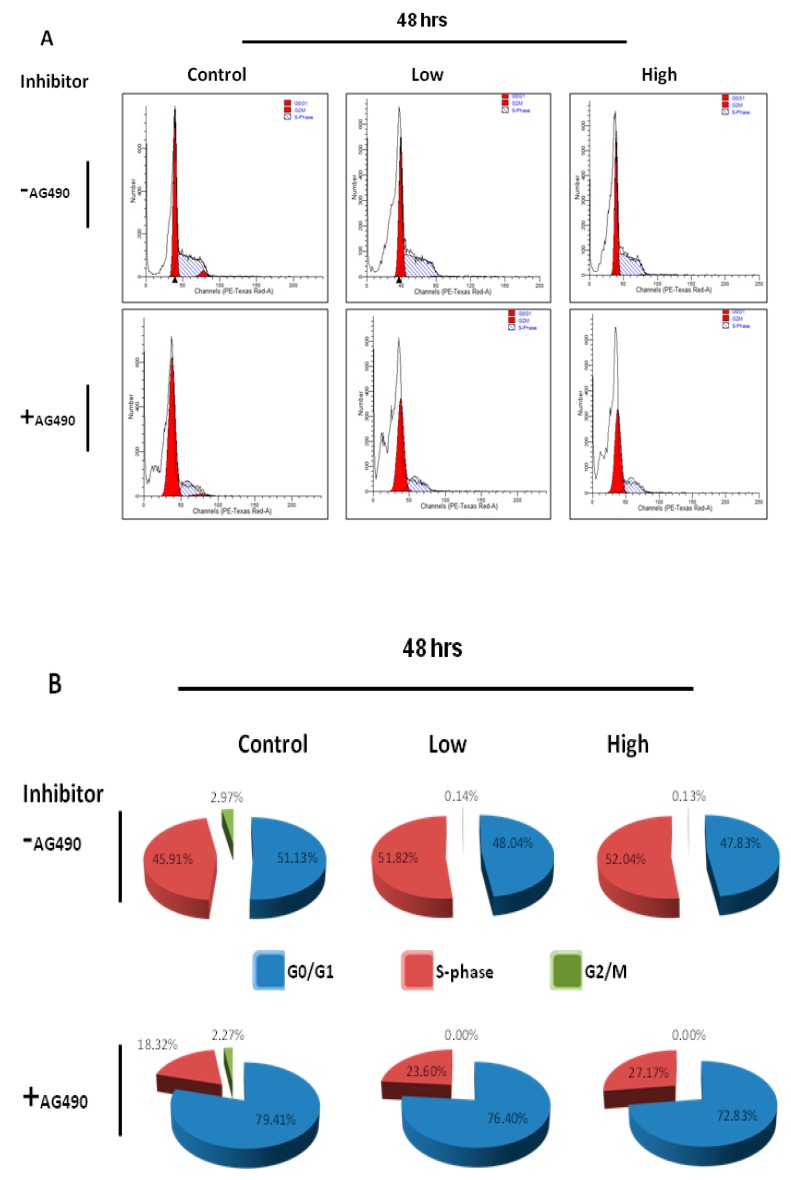
Effect of IL-6 concentration and JAK-STAT inhibitor on C2C12 cell cycling at 48 h. (**A**) Flow cytometric analysis of IL6-treated cells, with or without JAK-STAT inhibitor, to determine the effect of IL-6 concentration on C2C12 cell cycling was done after 48 h of incubation. Displayed are representative images (**B**) Summary of data shown in (**A**) of C2C12 cells after treatment with IL-6 and JAK-STAT inhibitor, AG490. The experiment was repeated three times. Results are presented as mean ± standard error of the mean (S.E.M). Statistical analysis: ANOVA and Bonferroni post hoc test.

To reveal mechanistic pathways through which IL-6 might affect C2C12 characteristics, the activation of the JAK-STAT pathway in IL-6-treated cells was examined. The activation of JAK-STAT signaling was examined in C2C12 cells via the use of antibodies against p-STAT3. All IL-6 concentrations (high, medium and low IL-6 levels) resulted in early induction of STAT3 phosphorylation, shown 5 min after addition of IL-6 (Figure 5A,B). Only high IL-6 concentration (10 ng/mL) appeared to prolong phosphorylation of STAT3 up to 10 min but no longer between 15 and 30 min (Figure 5A,B). Since treatment of C2C12 cells with low and medium IL-6 concentrations exhibited similar effect on cell cycle and associated proteins, low IL-6 concentration (10 pg/mL) and high IL-6 concentration (10 ng/mL) were chosen for further comparative experiments. To verify that effects of IL-6 are indeed mediated by STAT3 phosphorylation, responses were assessed in the presence of a JAK2-STAT3 inhibitor, AG490. AG490 was added to cultured C2C12 cells in the 48 h experiment with every media change (Figure 5C,D). Similar stocks of C2C12 cells were used for both inhibitor and no inhibitor experiments. Addition of STAT3 inhibitor resulted in reduced STAT3 phosphorylation compared to controls, irrespective of the IL-6 concentration used at the beginning of the 48 h experiment (Figure 5C). Finally, addition of the inhibitor resulted in decreased PCNA, MyoD and myogenin levels in IL-6-treated cells compared to controls at the end of the 48 h experiment (Figure 5D,E). At the end of the 48 h incubation, the JAK2-STAT3 inhibitor, AG490, resulted in more cells being in the G0/G1phase with a consequential decrease in cells in the S-phase for both low and high IL-6 concentrations (Figure 6A,B). The STAT3 inhibitor completely inhibited cell division in both low and high IL-6 concentration-treated experiments (Figure 6A,B).

### 2.2. Primary Human Myoblasts (PHM) Characterisation

The C2C12 mouse myoblast cell line is one of the most used muscle-derived progenitor cell lines for in vitro experimentation [80]. C2C12 cells have enhanced and rapid proliferation capacity and differentiate into myofibers effectively [80]. Isolated primary myoblasts have a higher sarcomere assembly than C2C12 under both proliferation and differentiation conditions [79]. This suggests that primary myoblasts represent the characteristics of the skeletal muscle more accurately than C2C12 cells/myotubes [78]. In order to fully elucidate the effect of IL-6 on human myogenesis and the signaling cascades involved, we therefore used primary human myoblasts in further investigations.

**Figure 7:**
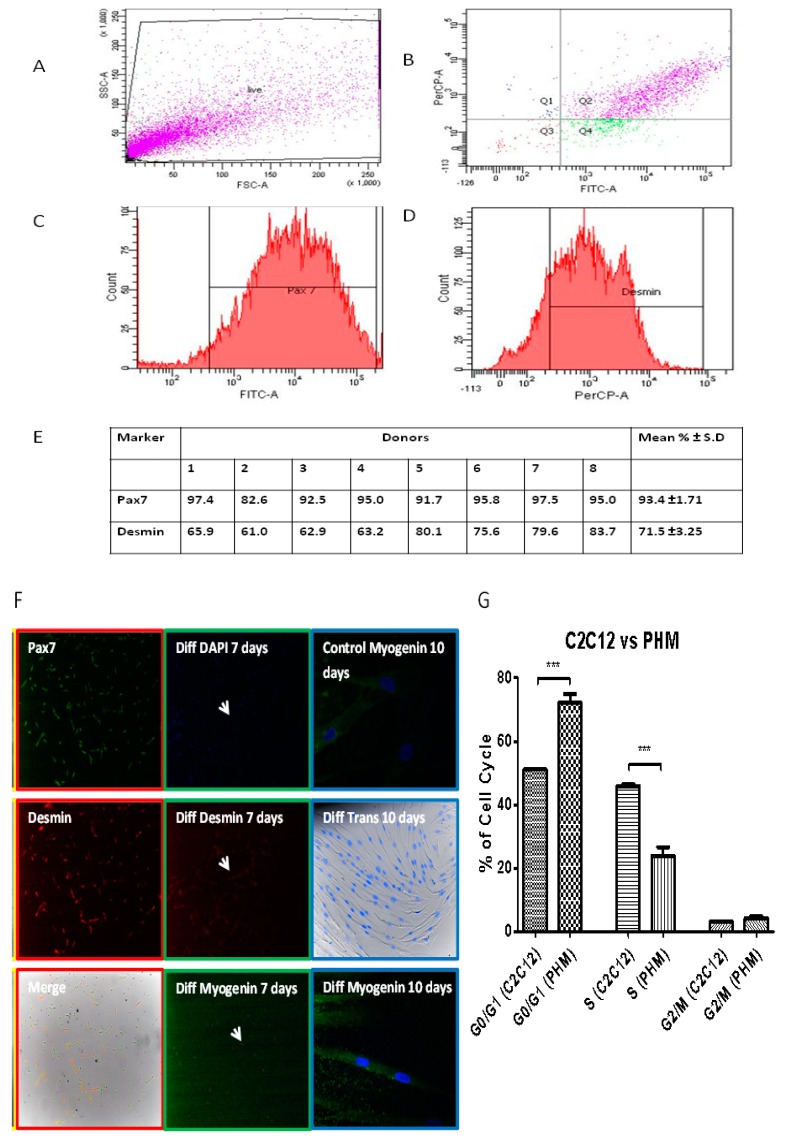
The phenotypic characterization of primary human myoblasts. (**A**–**D**) Flow cytometric analysis of Primary Human myoblasts (PHMs) was performed as described in Materials and Methods section. Average of cells (%) staining positive for Pax7 and desmin are shown. (**E**) Several preparations of the PHMs were analyzed and shown is representative of eight donors. Results are shown as mean ± S.D. (**F**) Red outlined column (first column): Preliminary characterization of isolated cells as performed to ascertain whether the culture generated contained myoblasts: Pax7 (green), desmin (red) (fluorescent microscopy at 100× magnification). Green outlined Column (second column): PHMs were exposed to differentiation inducing conditions for 7 days and stained via immunofluorescence for desmin (red), myogenin (green) and nuclei (blue). White arrows indicate multinucleated myofibres. Blue outlined Column (third column): PHMs were able to preserve their differentiation ability after long periods of sub-culturing. Cells were maintained and passaged for two months (imaged by confocal microscopy (myogenin) at 400× magnification or fluorescent microscopy (Trans) at 200× magnification). (**G**) Comparison of C2C12 and PHMs. Cells were analyzed through flow cytometry and percentages of cells in each cell cycle stage quantified. * *p* < 0.05; ** *p* < 0.01; *** *p* < 0.001.

Primary human myoblasts (PHMs) were isolated using the micro-explant technique. Preliminary characterization of isolated primary human myoblast cells was done through immunofluorescence staining for Pax7 and desmin (Figure 7A–F). The myogenic differentiation potential of the isolated cells was evaluated by exposure to differentiation inducing conditions for 7 days and immunostaining performed for desmin and myogenin. PHM expressed Pax7 and desmin and as expected phenotypic characterization of PHM showed that expression of Pax7 was in ≥ 90% of the cell population (Figure 7A–E). Furthermore, multinucleated fibers appeared after 7 days of incubation in differentiation media (Figure 7F). PHMs stained positively for the terminal differentiation transcription factor myogenin and the sarcomere specific protein desmin after incubation under differentiation inducing conditions (Figure 7F). The data show that isolated PHMs were able to preserve muscle phenotype even after long periods of sub-culturing. A comparison of C2C12 cells versus PHMs showed distinct differences in cell cycle profiles, justifying the choice of using primary human myoblasts over C2C12 cells to study human myogenesis (Figure 7G). PHMs showed increased number of cells in G0/G1 phase prior to IL-6 treatment and decreased number of cells in S phase compared to C2C12 cells (Figure 7G). This is however expected as primary cells are known to have reduced proliferative capabilities compared to immortalized cells.

### 2.3. High IL-6 Concentration Induced Myogenic Differentiation in Primary Human Myoblasts (PHMs)

**Figure 8:**
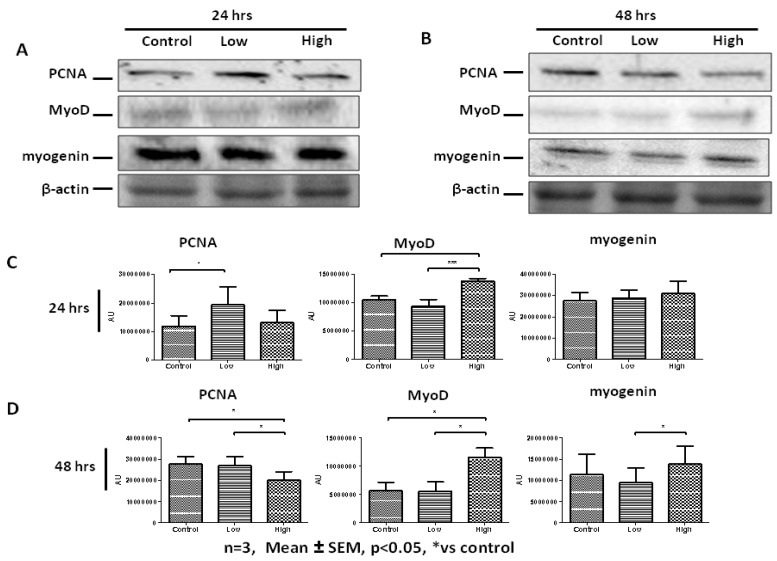

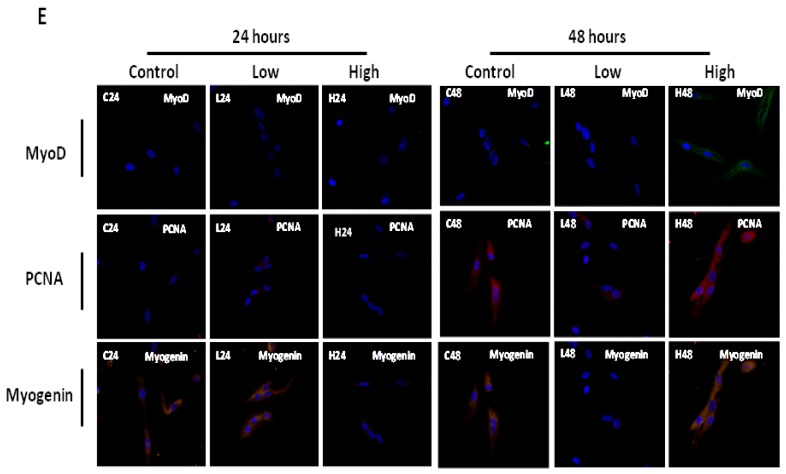
Concentration- and time-dependent effects of low and high IL-6 concentrations on PCNA, MyoD and myogenin expression in PHMs. (**A**) Western blot determination of PCNA, MyoD and myogenin protein levels 24 h after treatment with low and high IL-6 concentrations. (**B**) Western blot determination of PCNA, MyoD and myogenin protein levels 48 h after treatment with low and high IL-6 concentrations. (**C**) Quantification of PCNA, MyoD and myogenin protein levels as shown in (**A**). (**D**) Quantification of PCNA, MyoD and myogenin protein levels as shown in (**B**). (**E**) Immunofluorescence staining for PCNA, MyoD and myogenin protein in PHMs after treatment with low and high IL-6 for 24 and 48 h. The experiment was repeated three times. Immunofluorescence images were taken at 100× magnification. Results are presented as mean ± standard error of the mean (S.E.M). Statistical analysis: ANOVA and Bonferroni post hoc test. * *p* < 0.05; *** *p* < 0.001.

To evaluate IL-6-mediated changes in PHMs, cells were treated with low and high IL-6 concentrations for 24 and 48 h followed by determination of PCNA and myogenic regulatory factors (MRFs), MyoD and myogenin. Western blot analysis shows that at 24 h low IL-6 concentration caused significant increases in PCNA levels compared to controls (Figure 8A,C). At the same time point, low IL-6 had essentially no effect on MyoD expression compared to controls (Figure 8A,C). In contrast, treatment of PHMs with high IL-6 concentration resulted in increased MyoD levels compared to control cells at the 24 h time point (Figure 8A,C). Immunofluorescence analysis largely substantiated these findings (Figure 8E). After 24 h of incubation, low IL-6 concentration caused increased PCNA expression (Figure 8E). High IL-6 concentration-treated cells displayed MyoD and myogenin levels similar to controls, whilst PCNA was decreased.

Western blot analysis showed that further incubation of cells with high IL-6 concentration up to 48 h resulted in significant decrease in PCNA levels (Figure 8B,D). High IL-6 concentration caused significant increase in both MyoD and myogenin levels at 48 h (Figure 8B,D). These results are different from those obtained at 24 h. Thus IL-6 display time-dependent effects in PHMs. More importantly, increase in myogenin expression is a late event (48 h) after IL-6 exposure whilst increase in MyoD expression occurs early (24 h). The increased expression of MyoD early on before differentiation is in agreement with the myoblast phenotype and the order of events during myogenesis. Myogenic regulatory factors were clearly affected more in PHMs than in C2C12 cells, especially MyoD. Immunofluorescence data substantiated the western blot analysis, with the high IL-6 concentration markedly increasing the levels of MyoD and myogenin at the 48 h time point (Figure 8E). Thus, high IL-6 concentration favors differentiation over proliferation. Terminal differentiation of myoblasts, as shown by increased myogenin expression, induced by high IL-6 concentration occurred concomitantly with a decrease in PCNA expression.

### 2.4. IL-6 Concentration-Dependent Activation of JAK2-STAT3 Signaling in Human Myogenesis

**Figure 9:**
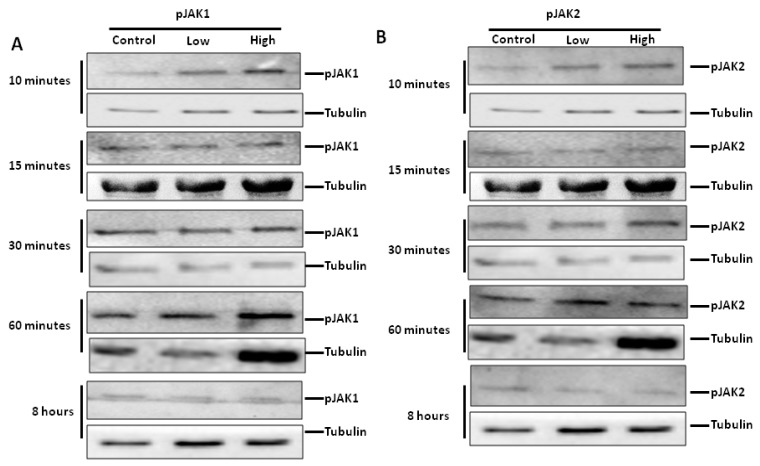

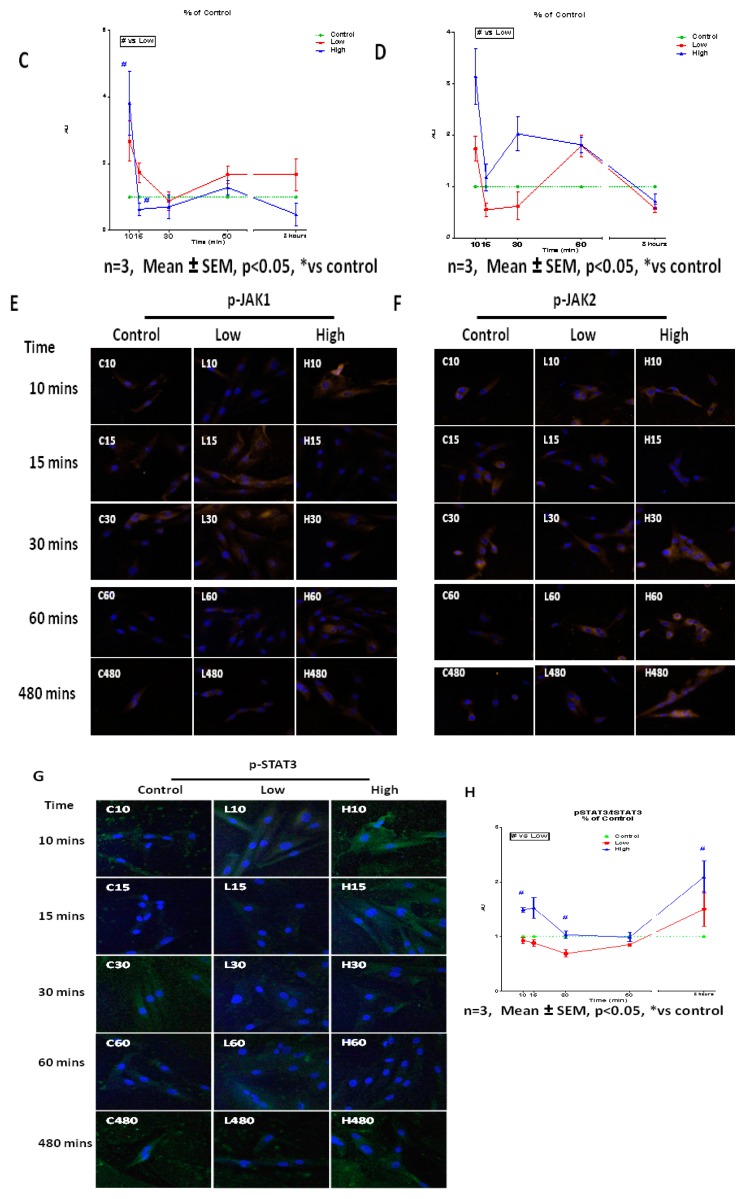
Effect of low and high IL-6 concentrations on JAK1 and JAK2 phosphorylation in PHMs. (**A**) Effect of low and high IL-6 concentration on JAK1 phosphorylation over 8 h. (**B**) Effect of low and high IL-6 concentration on JAK2 phosphorylation over 8 h. (**C**) Quantification of p-JAK1 as shown in (**A**). (**D**) Quantification of p-JAK2 as shown in (**B**). (**E**) Immunofluorescence staining for p-JAK1 after treatment of PHMs with low and high IL-6 concentration over 8 h. (**F**) Immunofluorescence staining for p-JAK2 after treatment of PHMs with low and high IL-6 concentration over 8 h. (**G**) Immunofluorescence staining for p-STAT3 after treatment of PHMs with low and high IL-6 concentration over 8 h (**H**) Quantification of p-STAT3 levels as shown in (**G**). The experiment was repeated three times. All immunofluorescence images were taken at 100× magnification. Results are presented as mean ± standard error of the mean (S.E.M). Statistical analysis: ANOVA and Bonferroni post hoc test. * *p* < 0.05; ** *p* < 0.01; *** *p* < 0.001.

Given the comprehensive analysis of cell cycle and myogenic regulatory factors in C2C12 cells, further investigation using PHMs focused on the role played by JAK-STAT signaling in IL-6-mediated behavior of primary human myoblasts. PHMs were treated with low and high concentrations of IL-6 and evaluation of the signaling pathways activated was done. In PHMs, activation of the JAK-STAT signaling pathway was followed up to 8 h after addition of IL-6, through evaluation of p-JAK1, p-JAK2 and p-STAT3 levels. Time points included 10, 15, 30, 60 min and 8 h. Figure 9 presents results on some time points as well as the effects of IL-6 concentrations. Without IL-6, there were no changes in p-JAK1 or p-JAK2 at any time points.

Addition of IL-6 activated both JAK1 and JAK2 in a concentration -dependent fashion (Figure 9A–D). Low IL-6 concentration resulted in significantly increased phosphorylation of JAK1 (Figure 9A,C). An initial increase in p-JAK1 due to high IL-6 concentration soon dissipated (Figure 9A,C). In contrast, low IL-6 concentration was able to maintain increased p-JAK1 levels over a longer time, up to one hour (Figure 9A,C). Treatment of primary human myoblasts with low IL-6 concentration resulted in significant decrease in p-JAK2 compared to controls (Figure 9B,D). In contrast to low IL-6 concentration, high IL-6 concentration resulted in significant increase in p-JAK2 and this increase was maintained over 8 h (Figure 9B,D). Immunofluorescence analysis substantiated western blot analysis, albeit with minor visual differences (Figure 9E). These minor differences might be borne out of the fact that confocal microscopy is qualitative whilst western blot analysis shows quantitative differences. In summary, low IL-6 concentration activated the phosphorylation of JAK1 whilst high IL-6 concentration activated the phosphorylation of JAK2.

JAKs are known to phosphorylate STATs. This concentration-dependent activation of the JAKs may have affected STATs phosphorylation. Treatment of PHMs with low IL-6 concentration did not affect p-STAT3 levels with levels predominantly similar to or lower than controls up to 60 min (Figure 9G,H). At 8 h the levels of p-STAT3 had increased significantly. High IL-6 concentration however, caused significant increase in p-STAT3 levels at early time points up to 30 min of incubation and after 60 min of incubation remained significantly higher (Figure 9G,H).

**Figure 10:**
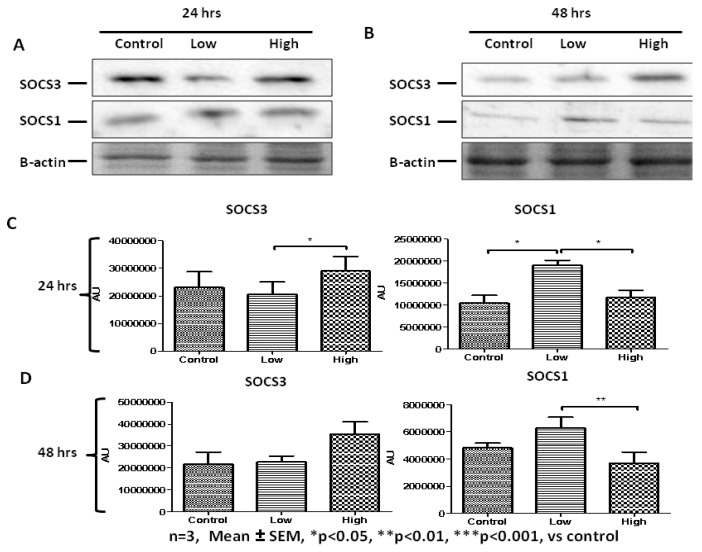

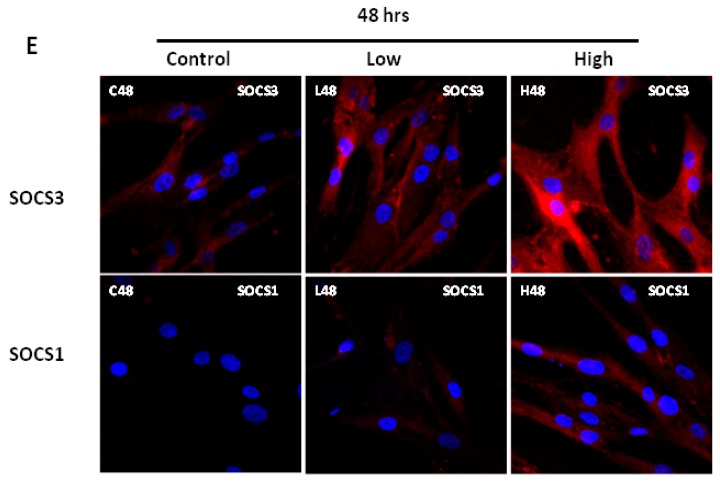
Concentration- and time-dependent effects of low and high IL-6 concentrations on SOCS1 and SOCS3 protein levels in PHMs. (**A**) Western blot determination of SOCS3 and SOCS1 protein levels 24 h after treatment with low and high IL-6 concentrations. (**B**) Western blot determination of SOCS3 and SOCS1 protein levels 48 h after treatment with low and high IL-6 concentrations. (**C**) Quantification of SOCS3 and SOCS1 protein levels as shown in (**A**). (**D**) Quantification of SOCS3 and SOCS1 protein levels as shown in (**B**). (**E**) Immunofluorescence staining for SOCS3 and SOCS1 after treatment of PHMs with low and high IL-6 concentration for 48 h. The experiment was repeated three times. Immunofluorescence images were taken at 100× magnification. Results are presented as mean ± standard error of the mean (S.E.M). Statistical analysis: ANOVA and Bonferroni post hoc test. * *p* < 0.05; ** *p* < 0.01.

We hypothesized that regulators of the JAK-STAT pathway are likely to come into play at later stages of the IL-6-mediated changes in cell behavior. We therefore evaluated the levels of SOCS1 and SOCS3 after addition of IL-6 for 24 and 48 h. After 24 h of incubation, the data show that low IL-6 concentration resulted in significantly decreased SOCS3 levels but increased SOCS1 levels compared to both controls and high IL-6 concentration (Figure 10A,C). High IL-6-treated cells displayed similar levels of both SOCS1 and SOCS3 compared to controls (Figure 10 A,C). After 48 h incubation, low IL-6 concentration showed elevated SOCS1 levels whilst high IL-6 concentration resulted in elevated SOCS3 levels (Figure 10B,D). Immunofluorescence analysis confirmed these results with minor differences (Figure 10E). Considering data on IL-6 effects on myogenic regulatory factors acquired from C2C12 cells and presented earlier, these data in PHMs suggest that elevated levels of SOCS3 are associated with cell differentiation. Overall, the data demonstrate that IL-6 activates the JAK-STAT signaling pathway and this can induce myoblast proliferation or differentiation depending on the JAK-STAT components involved and that IL-6 induces regulatory feedback.

### 2.5. IL-6 Receptor Regulation in Response to IL-6 Treatment

**Figure 11:**
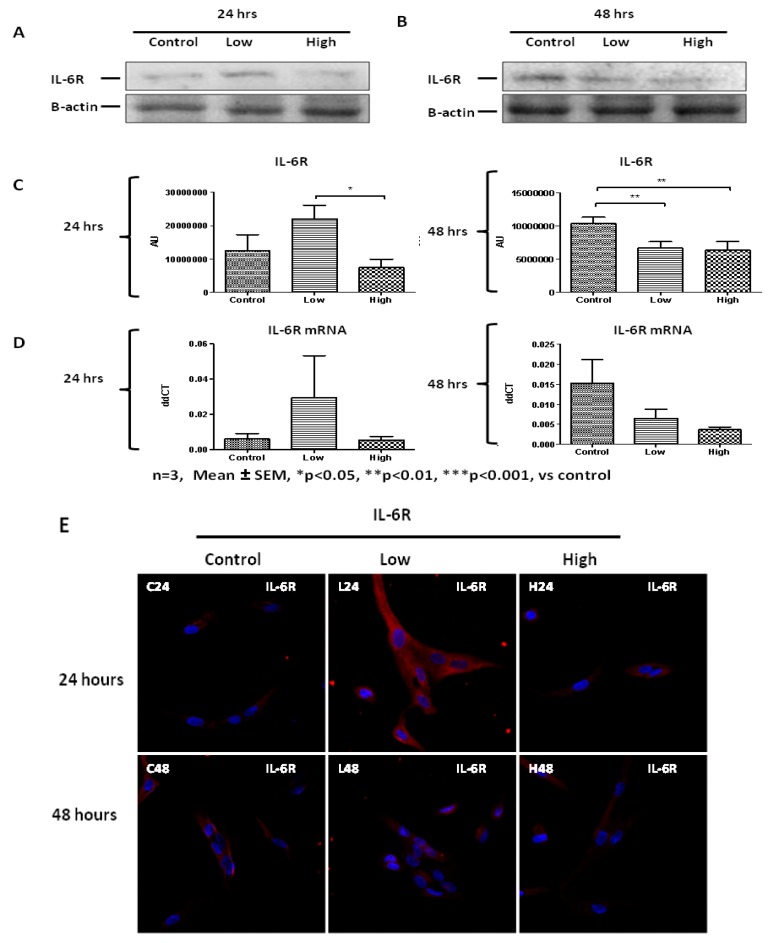
Concentration- and time-dependent effects of low and high IL-6 concentrations on IL-6R expression in PHMs. (**A**) Western blot determination of IL-6R protein levels 24 h after treatment with low and high IL-6 concentrations. (**B**) Western blot determination of IL-6R protein levels 48 h after treatment with low and high IL-6 concentrations. (**C**) Quantification of IL-6R protein levels as shown in (**A**). (**D**) Quantification of IL-6R mRNA levels after treatment of cells with low and high IL-6. (**E**) Real time PCR analysis of IL-6R mRNA transcripts 24 h after treatment of PHMs with low and high IL-6 concentration. (**E**) Real time PCR analysis of IL-6R mRNA transcripts 48 h after treatment of PHMs with low and high IL-6 concentration. (**E**) Immunofluorescence staining for IL-6R after treatment of PHMs with low and high IL-6 concentration for 24 (top panel) and 48 h (lower panel). The experiment was repeated three times. Immunofluorescence images were taken at 100× magnification. Results are presented as mean ± standard error of the mean (S.E.M). Statistical analysis: ANOVA and Bonferroni post hoc test. * *p* < 0.05; ** *p* < 0.01.

The transmembrane complex of IL-6 and its receptor IL-6R, allows IL-6 to bind to gp130 subunits of the receptor. Given the relatively long exposure of the PHMs to IL-6 in the media, an investigation was done on the effect of IL-6 on expression of IL-6 receptor in human primary cells. Indeed, IL-6R protein levels were altered by exposure to IL-6 (Figure 11). These alterations exhibited both positive feedback and downregulation at different times and with different concentrations of IL-6. At the 24 h time point western blot data show that low IL-6 concentration resulted in an upregulation of its receptor, IL-6R (Figure 11A,C). On the contrary, high IL-6 concentration resulted in no change in IL-6R (Figure 11A,C). After 48 h of incubation with IL-6, IL-6R levels were downregulated by treatment with both low and high IL-6 concentrations (Figure 11B,C). Thus, the upregulation of IL-6R protein was not sustained after 48 h at which time both concentrations had protein levels significantly below basal levels, probably indicating negative feedback (Figure 11A–C). RT PCR analysis of IL-6R transcripts also confirmed the results obtained via western blot indicating that this was an influence at the level of transcription factors (Figure 11D). Immunofluorescence data mirrored the protein data obtained (Figure 11E).

## 3. Discussion

**Figure 12:**
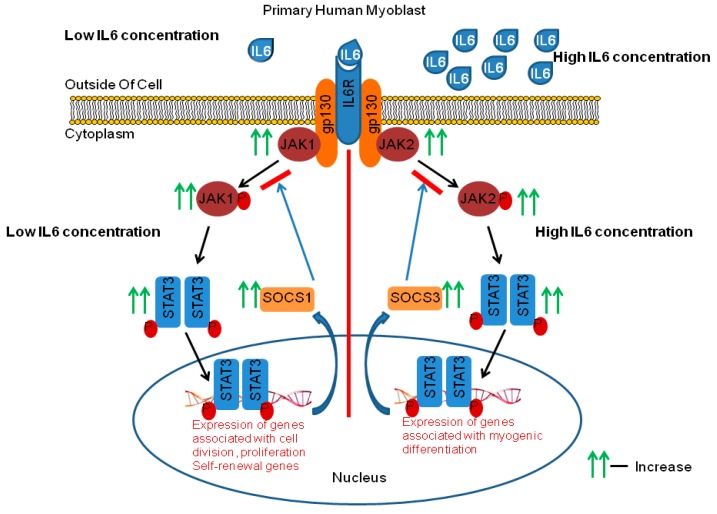
Schematic summary of IL-6 induced effects on the JAK-STAT pathway and its main regulator, SOCS. Low IL-6 concentration depicted on the left side and high IL-6 concentration on the right. Green arrows indicate findings of this study.

The main findings of this study were that IL-6 has concentration- and time-dependent effects on both C2C12 and primary human myoblasts, with evidence that low IL-6 concentration induces proliferation whilst high IL-6 concentration induces differentiation. Taking combinations of results together, this study shows firstly that low and medium IL-6 concentrations promoted both C2C12 and PHMs proliferation via upregulation of PCNA protein levels and decreased levels of myogenic regulatory factors, MyoD and myogenin. Low and medium IL-6 concentrations also activated the JAK1-STAT3 cascade to promote myoblasts’ proliferation. In contrast, high IL-6 concentration decreased PCNA protein levels and promoted myoblasts’ differentiation through increasing the levels of myogenic regulatory factors, MyoD and myogenin. Also, in contrast, high IL-6 concentration activated the JAK2-STAT3 cascade. Secondly, inhibition of the JAK-STAT3 cascade in the C2C12 cells confirmed and illuminated several findings such as myoblasts’ reduced proliferation in the presence of AG490, a JAK-STAT3 inhibitor, a finding also associated with reduced expression of PCNA, despite the presence of low IL-6. Downregulation of PCNA means that PCNA is a STAT3 target gene. In contrast, despite the presence of high IL-6, inhibition of the JAK2-STAT3 cascade was shown to prevent early differentiation of myoblasts, through the repression of genes associated with myogenic differentiation (MyoD and myogenin). Thus, STAT3 plays a central role in controlling myoblast proliferation and differentiation. Thirdly, another significant observation was the IL-6-mediated increase in the expression of JAK-STAT3 downstream genes such as *SOCS1* and *SOCS3* and *IL-6R*. Specifically, JAK1-STAT3 was associated with increased SOCS1 expression, whilst JAK2-STAT3 was associated with increased SOCS3 expression. Low and high IL-6 concentrations affected IL-6R differently, with low IL-6 concentration increasing IL-6R protein levels whilst high IL-6 concentration reduced IL6-R levels (findings are summarized in Figure 12). These findings provided insights into mechanisms controlling myoblast fate in skeletal muscle. Finally, the time-dependent effects were seen specifically for PCNA and myogenin. Specifically, the effect of high IL-6 on PCNA reduction and myogenin increases took 48 h to occur, whereas with low IL-6 the increase in PCNA was rapid and evident at 24 h. Below we discuss our results in the context of effects of IL-6 on different cell types and under different conditions as determined by others. The discussion then focuses on the implications of our data on skeletal muscle physiology and pathological conditions.

Muscle injury and debilitating skeletal muscle diseases cause muscle satellite cells to undergo a process of activation, proliferation and finally differentiation. Several cytokines are thought to be involved in the activation of satellite cells including LIF [28,49,83]. LIF is a member of the IL-6 superfamily. Early differentiation is blocked until the pro-proliferation signals are reduced or eclipsed by pro-differentiation signals. The order of events as displayed by our data during IL-6-mediated differentiation of primary human myoblasts is in line with published data. It is known that MyoD is expressed early on before differentiation [35,38,41,45,74,84,85,86]. The expression of myogenin finally shows when myoblasts are fully committed to differentiation and is therefore a late event. Only high IL-6 induced myogenin in the current study. Once cells are differentiating, proliferation is reduced and here, the data clearly demonstrate decreases in PCNA levels once myoblasts start expressing myogenin, a terminal differentiation marker.

Several signaling pathways have been associated with the process of myogenesis and these include the MEK-ERK, PI3K-Akt, Notch and the JAK-STAT pathways [74,85,87,88]. In this study, specific focus was on the JAK-STAT signaling pathway, since previous work showing that the JAK-STAT pathway predominates over other pathways in terms of mediating IL-6 effects [72,89,90]. However, how this signaling pathway is activated by IL-6 during different phases of myogenesis has not been fully elucidated. Also, other studies did not consider concentration or duration of exposure of cells to IL-6 and the study by Hassan for example was in hepatic disorders [89]. In agreement with published data, our work reveals that JAK1-induced signaling operates mainly in the proliferation stage of muscle regeneration [87,91]. The interaction between different members of the JAK-STAT pathway results in context-dependent regulation of cellular processes [74,92,93,94]. In our study we show the involvement of JAK1/2, STAT3 and SOCS1/SOCS3 in myoblasts proliferation and differentiation. Several murine studies and studies involving macrophages showed that STAT3 can direct myoblasts’ behavior in a context-dependent fashion, with the involvement of specific JAKs [45,74,92,95]. A study by Sun and colleagues concluded that there are several JAK-STAT cascades that are activated in myoblasts and these might have different effects on proliferation and differentiation [87]. STAT3 ablation has been shown to result in p-STAT3, MyoD1 and myogenin downregulation before and our data confirmed this result in a context with somewhat more physiologically relevant IL-6 interventions [96]. Future studies focusing on delineating how phosphorylated STAT3 can activate different genes in response to different IL-6 concentrations must be undertaken.

Several studies have evaluated the effect of IL-6 on a variety of cells *in vitro*. Kurosaka and Machida evaluated the effects of IL-6 on rat primary muscle satellite cell proliferation [97]. The authors used IL-6 concentrations similar to the ones used in the current study. The authors used IL-6 concentrations ranging from 10 pg/mL up 100 ng/mL and they showed that 10 pg/ ml–1 ng/mL IL-6 induced satellite cell proliferation [97]. These results are similar to our data showing that low (10 pg/mL) and medium (100 pg /mL) IL-6 concentrations induced PCNA protein levels. Our data showing high (10 ng/mL) IL-6 concentration increased p-STAT3 activation resulting in primary myoblast differentiation, agree with data obtained by the same authors. Results from Kurosaka and Machida show that even 1 ng/mL IL-6 increased p-STAT3 activation [97]. However, Kurosaka and Machida showed that IL-6 induced satellite cell proliferation via the JAK2-STAT3 pathway. Our data showed that it is the JAK1-STAT3 pathway that is induced in primary myoblasts by low IL-6 concentration. Although in our study we did not use 1 ng/mL IL-6, our observations that medium (100 pg/mL) and low (10 pg/mL) IL-6 promote myoblasts proliferation, appear to agree with results obtained by Kurosaka and Machida when they used 1 ng/mL IL-6. One of our main aims was to try to cover a wide range of IL-6 concentrations, because several studies performed in animals and humans on the role of IL-6 in myogenesis, exercise-induced hypertrophy and aging, have delivered contrasting results [55,59,98,99]. Therefore, our study adds to our understanding of the human myogenesis process and how it is influenced by IL-6.

The JAK-STAT signaling pathway has several classes of regulators. These include the suppressors of cytokine signaling (SOCS) and the protein tyrosine phosphatases (PTPs) [74,91]. Several members of the SOCS family are known to be involved in myoblast behavior [74,100]. Specifically, SOCS1 and SOCS3 have been implicated in myoblasts proliferation and differentiation [91]. In the aforementioned study, Diao and colleagues showed that both SOCS1 and SOCS3 promote myogenic differentiation of mouse myoblasts in response to LIF [91]. However, our study demonstrates the involvement of SOCS1 and SOCS3 in primary human myoblasts proliferation and differentiation, respectively. Despite being members of the same superfamily, LIF and IL-6 may operate differently. In addition, it is important to note that our study involves the use of primary human myoblasts whilst the above study used mouse myoblasts. Other contrasting data having been published with some studies showing that SOCS3 is involved in cell differentiation as we show in this study [101] and others showing that SOCS3 is actually involved cell proliferation [97,100,102]. These contrasting findings may be due to concentrations or exposure time of the factors influencing SOCS3. Indeed, only treatment of primary human myoblasts in the current study with high IL-6 concentration resulted in significant increase in SOCS3 expression. When using IL-6 concentrations comparable to those used in the current study, Senn and colleagues demonstrated that IL-6 can induce SOCS3 expression in hepatocytes [103].

In the context of skeletal muscle, an increase in IL-6R mRNA has been seen in regenerating myotubes [82,104] which shows a possible role amongst the many factors regulating myoblast proliferation or differentiation. Wada and colleagues showed that anti-IL-6R antibody can be used to promote skeletal muscle regeneration in mice [82]. That study showed that low IL-6 concentration caused increased IL-6R protein levels. In contrast, high IL-6 concentration in the current study resulted in greater activation of JAK2-STAT3 and could be accountable for the down-regulation of the IL-6R at 24 and 48 h. Extended signaling through the IL-6R in IL-6-treated cells could trigger an auto-regulation feedback response. Alteration in receptor regulation between low and high IL-6 concentration could be responsible for their seemingly alternate roles in proliferation and differentiation. These speculations require further experiments on the physical interactions between the IL-6R and the JAKs to confirm these possible explanations. Physiological conditions of sustained high IL-6 in circulation in humans include cachexia [105,106,107,108]. Therefore, the data presented here could provide valuable information on possible treatment avenues for conditions affecting muscle, such as cachexia or myopathy. Low IL-6 concentration may induce IL-6R expression to promote sensitivity to the cytokine, whilst high IL-6 concentration reduces IL-6R expression to protect against too high IL-6 signaling. It is known that a soluble form of the IL-6 receptor is also secreted and IL-6 may signal via both the soluble and the membrane-associated form of IL-6R [45,74,92]. The possible involvement of this soluble receptor requires further investigations that are beyond the scope of this study. Produced as a result of alternative splicing and limited proteolytic processing, the soluble IL-6R is found in several body fluids [69,72]. The complex formed as a result of the coming together of IL-6 and the soluble IL-6R bind to gp130 homodimer on cells lacking the IL-6 receptor and has been suggested to amplify the activities and effect of IL-6 [69,72,73]. It is plausible therefore to postulate that the effects of IL-6 observed in this study might have involved the soluble IL-6R. Specifically, when the IL-6 concentration to which cells were exposed was high, both receptors may have been involved in promoting differentiation. IL-6R dynamics are also more complex than the comparison of IL-6 binding to surface-based and soluble receptors. For example, turnover of both receptors occurs under normal conditions and IL-6 bound to surface receptors may not remain on the surface but internalize. Studies to elucidate this phenomenon should be done in future in the context of dose and response time.

Whilst the C2C12 cell line is useful to study muscle biology and processes such as myogenesis and metabolism, the cell line has several limitations. Firstly, higher passage number cells have reduced proliferative and differentiation capabilities. Also, C2C12 cells lack the cell cycle regulator, p16 [80]. The effect of low IL-6 concentration on PCNA in C2C12 cells was still large at 48 h, suggesting that the immortalized cell line had a greater propensity to remain in proliferation mode. Our study confirmed that primary myoblasts proliferate slower than immortalized cells and differentiation was slightly delayed in primary myoblasts compared to C2C12 cells. However, high IL-6 concentration increased myogenin expression at 48 h much more prominently in C2C12 cells. All these responses were inhibited with addition of the JAK-STAT3 phosphorylation inhibitor, AG490, confirming that the effects were indeed due to IL-6.

Thus, our study demonstrates, for the first time in primary human myoblasts, that the level of IL-6 and the consequential activation of the JAK-STAT pathway can determine the proliferation or differentiation of human myoblasts in vitro, making IL-6 in the muscle microenvironment a critical cytokine in the context of regeneration. The critical balance between proliferation and differentiation of myoblasts is important. Here we show that the duration of exposure to IL-6 plays an important role. Importantly, the data suggests that IL-6 can modulate myoblast behavior in a paracrine manner, as exogenous addition of IL-6 induced several changes to the myoblasts. Our study has confirmed previous results and extended the knowledge of the role of IL-6 concentration on muscle regeneration. Mechanistic data presented here provide new directions in understanding the effect of cytokines on myoblasts proliferation and differentiation.

## 4. Materials and Methods

### 4.1. IL-6 Treatment Protocol

Cultured C2C12 cells and primary human myoblasts were treated with a range of IL-6 (PeproTech, Rehovot, Israel) concentrations ((low (10 pg/mL), medium (100 pg/mL) and high (10 ng/mL)) and a control group supplemented with PBS. In a bid to cover a wide range of IL-6 concentration and not to repeat what has been researched on, we chose the above IL-6 concentrations. Several studies, including Kurosaka and Machida [81,97] have used IL-6 within the same range. Standard media was removed from the wells and then standard media containing the above concentrations of IL-6, were added to cultured cells. This media change was regarded as time 0 h. Incubation was continued for 24 h after which cells were harvested for assays as described below. This was taken as time 24 h. For longer incubations, media was removed at 24 h and fresh IL-6 supplemented media was added to cultured cells for the next 24 h until the end of experiment (48 h). The replacement of the media with fresh media (2 mL per well in a 6-well plate) supplemented with IL-6 was done to maintain the concentration of IL-6 within the media. After the specified incubation periods, cells were harvested and used in assays as described below. All chemicals used in the study were supplied by Sigma-Aldrich (Aston Manor, South Africa) unless otherwise stated. 

### 4.2. Cell Culture 

C2C12 cells were cultured in Dulbecco’s Modified Eagle Medium (DMEM, D5546) supplemented with 10% (*v*/*v*) Fetal Bovine Serum (FBS, GIBCO™, Paisley, Scotland) 1% (*v*/*v*) Penicillin/Streptomycin (PenStrep, P4333) and 6.8% (*v*/*v*) *L*-Glutamine (200 mM, G7513). The cells were kept at 37 °C in a humidified incubator with 5% CO_2_. Primary human myoblasts were cultured in a medium made up of Ham’s F10 Nutrient Mixture Medium (F10, N6013) supplemented with 20% (*v*/*v*) Fetal Bovine Serum (FBS, GIBCO™, Paisley, Scotland), 2% (*v*/*v*) Penicillin/Streptomycin (PenStrep, P4333), 0.1% (*v*/*v*) Gentamicin (GIBCO™, Paisley, Scotland, 50 mg/mL, 15750-060), 6.8% (*v*/*v*) *L*-Glutamine (200 mM, G7513) and 10 ng/mL rhFGF (Promega, Madison, WI, USA, G5071). Cells were passaged with Trypsin-EDTA once 70%–80% confluence was reached.

### 4.3. Isolation and Culture of Human Primary Myoblasts

The isolation and culture of human primary myoblasts was done based on published protocols with minor modifications [109,110,111]. Eight healthy male subjects were recruited who participated in light exercises not more than twice per week and were not on chronic medication. All subjects were Caucasian with an age range of 20–25 years with an average height of 179 ± 3 cm. The average weight of subjects was 77 ± 16 kg. Muscle was collected from each subject using a trephine biopsy needle. Informed consent was obtained using guidelines approved by Sub-committee C of the Research Committee of University of Stellenbosch (Ethics Number: N12/08/051,12 November 2012), following the principles of the Declaration of Helsinki. Participants completed a health history questionnaire and had no muscle injury history. A needle biopsy was performed by a qualified doctor on the *vastus lateralis* of the subjects to harvest the muscle samples. The muscle samples were dissected/minced into 1 mm^3^ pieces and transferred into a plate containing PHMPM. All cell culture growth surfaces were pre-emptively coated with an ECL cell attachment matrix (Millipore, 08-110, Darmstadt, Germany). The samples were maintained in a semi-conditioned media by removing half of the old media in each well and replacing it with fresh media at regular intervals. The human primary myoblasts started to migrate off the fibers approximately 10 days post transfer. Biopsies were removed from the wells at 14 days post transfer and the adherent cells were subjected to sub-culturing in the respective primary human myoblast media.

### 4.4. Human Primary Myoblasts Characterisation

Isolated human primary myoblasts were characterized by evaluation of Pax7 and desmin expression via flow cytometric analysis as described before. Cells were trypsinised and resuspended in cold PBS at approximately 1 × 10^6^ cells/mL. Cells were fixed and permeabilized in a 1:1 Methanol/Acetone solution for 10 min on ice. Washing was done using PBS and resuspension was done in 1% (*v*/*v*) bovine serum albumin (BSA). Cells were incubated in the presence of mouse anti-Pax7 (1:200) and rabbit anti-desmin (1:100) primary antibodies for 30 min at room temperature. Cells were washed, centrifuged and resuspended in 1% (*v*/*v*) BSA. Secondary antibodies (donkey anti-mouse Alexa 488 (1:200), donkey anti-rabbit IgG PerCP (1:100)) were added for 30 min at room temperature. Cells were centrifuged and resuspended in PBS and stored on ice until they were analyzed using the BD FACSAria™ Cell Sorter (BD Biosciences, San Jose, CA 95133 USA). To determine the myogenic differentiation potential of primary human myoblasts, cells were treated with differentiation media (DMEM, 2% horse serum). To evaluate terminal differentiation, myogenin levels were determined. Proliferation of primary human myoblasts was determined using trypan blue staining and cell counting.

### 4.5. Cell Cycle Analysis

Approximately 1 × 10^5^ cells were trypsinised and resuspended in citrate buffer. The pH of the buffer was adjusted to 7.60. Cells treated with different concentrations of IL-6 for different time periods were then processed for flow cytometric analysis in accordance with standard protocols with the aid of a DNA reagent kit (BD Biosciences CycleTest^TM^ Plus, 340242, San Jose, CA, USA). Cells were fixed with 40% methanol and then incubated with Propidium Iodide and this was used to stain DNA prior to flow cytometry. Cell cycle analysis was done using flow cytometry (BD FACSAria™ Cell Sorter, BD Biosciences, San Jose, CA 95133 USA). About 1 × 10^4^ cells were analyzed and cell cycle fractions were quantified with ModFitLT 3.0 (Verity Software).

### 4.6. Immunoblot Analysis

Immunoblotting was carried out as described in standard procedures. At the end of each treatment, C2C12 and primary myoblast cells were washed with PBS and lysed in RIPA buffer. Protease inhibitors were added to prevent protein degradation. The following were the protease inhibitors used—leupeptin (1 µg/mL; for lysosomal proteases), pepstatin A (1 µg/mL; for aspartic proteases); Aprotinin (1 µg/mL; for trypsin and chymotrypsin); phenylmethylsulfonyl fluoride (PMSF, 1 µM, for serine and cysteine); sodium fluoride (NaF, 1 µM, for acidic phosphatases) and sodium orthovanadata (1 mM, for tyrosine and alkaline phosphatases). Protein concentration was determined using the BCA assay. Proteins (10 µg) were then separated on 6%–10% sodium dodecyl sulphate polyacrylamide gel electrophoresis (SDS-PAGE) gels in the presence of B-mercaptoethanol. Transfer of proteins was done using a polyvinylidene difluoride (PVDF) membrane (Pall Corporation, Port Washington, NY, USA, #T010981). Membranes were blocked with 5% (*w*/*v*) non-fat milk powder dissolved in 1% (*v*/*v*) Tris buffered saline (TBS)-Tween buffer. Incubation with primary antibodies was done overnight at 4 °C. All primary antibodies utilized in this study are shown in Table 1. The membranes were washed twice with 1% (*v*/*v*) TBS-Tween and incubated with specific horseradish peroxidase (HRP)-conjugated secondary antibodies (BioRad, Hercules, CA, USA) for 1 h at room temperature with constant agitation. Secondary antibodies used in the study are shown in Table 2. Membranes were then washed twice with 1% (*v*/*v*) TBS-Tween buffer. Detection of proteins was done using Chemiluminescence substrate (SuperSignal West Femto Chemiluminescent Substrate, Thermo Scientific, Waltham, MA, USA, 23227) and images were taken using the ChemiDoc^TM^ MP Imaging System (Bio-Rad Laboratories, Hercules, CA, USA). Densitometric analysis was performed using Image Lab^TM^ Software (Bio-Rad Laboratories, Hercules, CA, USA). All experiments were performed at least twice.

### 4.7. Immunocytochemistry Staining 

Primary myoblasts and C2C12 cells were cultured on top of glass slides and treated with IL-6 as described above. Cells were fixed with 4% (*v*/*v*) paraformaldehyde for 15 min at room temperature. Slides were washed twice with PBS for 5 min. Cells were permeabilized with 0.50% (*v*/*v*) Triton X-100 (BDH Laboratories, Poole, UK, 306324N) in PBS for 15 min at room temperature. To prevent non-specific binding, cells were blocked using 20% (*v*/*v*) Donkey Serum (D9663) for 30 min at room temperature. Incubation with primary antibodies, diluted in 20% Donkey serum, was done overnight at 4 °C. Cells were then incubated with the appropriate secondary antibodies, diluted in donkey serum, for 60 min in the dark at room temperature. All primary and secondary antibodies used in the analyses are listed in Table 1 above and Table 3 below, respectively. Incubation with a nuclear stain (Bis Benzimide H33422 trihydrochloride, Sigma-Aldrich, Aston Manor, South Africa #B2261) was done for 15 min at room temperature. Slides were visualized by fluorescence (Olympus IX81 fitted with CellR® software, Waltham, MA, USA) or confocal microscopy (Carl Zeiss Confocal LSM 780 Elyra S1 fitted with Zen software, Jena, Germany). A minimum of three different images were obtained per each treatment condition.

### 4.8. RNA Preparation and RT-qPCR

Total RNA was isolated from cells using Trizol reagent (BioRad, California, USA) based on the procedure of Chomczynski and Sacchi [112]. The quality and quantity of the RNA was assessed using spectrophotometry and agarose gel electrophoresis. Total RNA from each sample was reverse transcribed using the First Strand cDNA Synthesis kit (Roche, Belmont, CA, USA, 04896866001) according to the manufacturers’ instructions. Random hexamers were added to the DNase treated RNA and was incubated in the thermal cycler for 10 min at 65 °C. Quantitative PCR was performed to detect IL-6R mRNA and monitored using the StepOnePlus™ System (Life Technologies, Carlsbad, CA, USA). cDNA samples from triplicate samples were analyzed using primers as shown in Table 4. Conditions for thermocycling were: 2 min at 50 °C, initial denaturation for 10 min at 95 °C, followed by 40 cycles of 15 s at 95 °C and 1 min at 60 °C and 72 °C for 20 s. The C_T_ value represents the target threshold which is the number of cycles necessary for the fluorescent signal generated to rise above the background levels. The C_T_ is inversely proportional to the number of mRNA transcripts, so a lower C_T_ would indicate higher mRNA transcripts. Relative gene expression was then computed for each sample in comparison with each corresponding control by using the ddC_T_ method. The amount of target mRNA was then finally calculated by the equation: amount of target = 2^−dd*C*T^ [113]. The *beta-2-microglobulin (B2M*) gene was used as the housekeeping gene.

### 4.9. Statistical Analysis

Statistical analysis was done using GraphPad Prism version 5. Data were expressed as means ± Standard error of the mean (S.E.M). ANOVA and the Bonferroni post hoc test were used to ascertain the statistically significant differences between controls and treated samples. Data: *n* = 3 observations in each experiment was 3. Each observation was done on myoblasts obtained from other donors. Statistical significance was accepted when * *p* < 0.05; ** *p* < 0.01; *** *p* < 0.001.

## 5. Conclusions

This study demonstrated that IL-6 play key roles in myoblasts proliferation and differentiation, with the myokine displaying both concentration and time-dependent effects on myoblasts fate. IL-6 activates different components of the JAK-STAT signaling pathway to influence the fate of myoblasts. Overall, our study has deepened our understanding of the role of IL-6 in myoblasts proliferation and differentiation. Furthermore, we have revealed that the IL-6/JAK/STAT/SOCS pathway is a promising therapeutic target in muscle wasting pathological conditions.

## Figures and Tables

**Table 1:** Primary antibodies utilized for western blotting and immunocytochemistry.

1°Antibody	Manufacturer	Species	Catalogue Number
MyoD	Santa Cruz	Mouse	SC32758
Myogenin	Santa Cruz	Mouse	SC12732
PCNA	Santa Cruz	Rabbit	SC7907
pSTAT3	Cell Signaling	Rabbit	9131
tSTAT3	Cell Signaling	Rabbit	9132
pJAK1	Santa Cruz	Goat	SC16773
pJAK2	Santa Cruz	Goat	SC21870
SOCS3	Santa Cruz	Rabbit	SC9023
SOCS1	Santa Cruz	Rabbit	SC9021
IL-6Rα	Santa Cruz	Rabbit	SC13947
β-actin	Cell Signaling	Rabbit	4967

**Table 2:** Secondary antibodies utilized for western blotting.

2°Antibody	Manufacturer	Description
Anti-mouse	Abcam	Goat HRP conjugated
Anti-rabbit	Santa Cruz	Goat HRP conjugated
Anti-goat	Abcam	Sheep HRP conjugated

**Table 3:** Secondary antibodies utilized for immunocytochemistry.

2°Antibody	Manufacturer	Species	Product Number
Alexa Fluor 488	Invitrogen	Donkey Anti-mouse IgG	A21202
Alex Fluor 546	Invitrogen	Donkey Anti-goat IgG	A11056
Alexa Fluor 594	Invitrogen	Donkey Anti-rabbit	A21207

**Table 4:** Primers utilized for quantitative polymerase chain reaction (qPCR).

Primer	Manufacturer	Primer Code
IL-6R	Applied Biosystems	Hs1075666_m1
Housekeeping (B2M)	Applied Biosystems	Hs00984230_m1

## Abbreviations

CD34	Cluster of Differentiation 34
FBS	Fetal Bovine Serum
HGF	Hepatocyte Growth Factor
IL-6	Interleukin 6
JAK	Janus kinase
LIF	Leukemia Inhibitory Factor
MEF	Myocyte Enhancer-binding Factor
MRFs	Myogenic Regulatory Factors
PCNA	Proliferating Cell Nuclear Antigen
PHM	Primary Human Myoblast
SOCS	Suppressor of Cytokine Signaling
STAT	Signal Transducer and Activator of Transcription
TGF-β	Transforming Growth Factor-β
